# Alterations in the innate immune system due to exhausting exercise in intensively trained rats

**DOI:** 10.1038/s41598-020-57783-4

**Published:** 2020-01-22

**Authors:** Sheila Estruel-Amades, Mariona Camps-Bossacoma, Malén Massot-Cladera, Francisco J. Pérez-Cano, Margarida Castell

**Affiliations:** 1 Secció de Fisiologia, Departament de Bioquímica i Fisiologia, Facultat de Farmàcia i Ciències de l’Alimentació, Universitat de Barcelona (UB), Barcelona, Espanya Spain; 2 Institut de Recerca en Nutrició i Seguretat Alimentària (INSA-UB), UB, Barcelona, Spain

**Keywords:** Quality of life, Experimental models of disease, Preclinical research

## Abstract

It is known that intensive physical activity alters the immune system’s functionality. However, the influence of the intensity and duration of exercise needs to be studied in more depth. We aimed to establish the changes in the innate immune response induced by two programmes of intensive training in rats compared to sedentary rats. A short training programme included 2 weeks of intensive training, ending with an exhaustion test (short training with exhaustion, S-TE). A second training programme comprised 5-week training including two exhaustion tests and three trainings per week. In this case, immune status was assessed before (T), immediately after (TE) and 24 h after (TE24) an additional final exhaustion test. Biomarkers such as phagocytic activity, macrophage cytokine and reactive oxygen species (ROS) production, and natural killer (NK) cell activity were quantified. S-TE was not enough to induce changes in the assessed innate immunity biomarkers. However, the second training was accompanied by a decrease in the phagocytic activity, changes in the pattern of cytokine secretion and ROS production by macrophages and reduced NK cell proportion but increased NK cytotoxic activity. In conclusion, a 5-week intense training programme, but not a shorter training, induced alterations in the innate immune system functionality.

## Introduction

Moderate exercise induces many beneficial effects on human health. In particular, it reduces the risk of developing many chronic disorders, such as cardiovascular disease^1^, metabolic syndrome^2^, type 2 diabetes^3^ and even cancer^4,5^. Furthermore, moderate exercise has an antioxidant effect^6^ and a positive influence on the innate immune system^7,8^. On the other hand, intense exercise can induce adverse effects on health, which enhance the risk of upper respiratory tract infections (URTI)^9,10^.

The observations regarding the amount and intensity of exercise and risk of illnesses have led to the hypothesis that their relationship fits a J-shaped curve^11^. This means that very low physical activity or no training is associated with a higher risk of illness compared to moderate activity or training load, whereas very high training loads are related to a higher risk of illness^10^. In other words, intensive exercise produces a decrease in the immune system functionality which makes the body more vulnerable to infectious agents. This period is known as an ‘open window’ to pathogens^12,13^.

The innate immune system is the first line of organism defence against pathogens. Neutrophils, macrophages and natural killer (NK) cells are the major cellular type of innate immunity susceptible to being altered by exercise. Neutrophils are phagocytic cells able to destroy bacteria by means of the release of enzymes and through the secretion of reactive oxygen species (ROS). Monocytes and macrophages are also phagocytic cells able to secrete a wide range of cytokines, mainly with pro-inflammatory properties^14,15^. NK cells are lymphocytes found in small proportions in blood and lymphoid tissues. Nevertheless, due to their cytotoxic activity and ability to fight against virus-infected cells, NK cells play an important role in innate immunity.

It has been shown that blood neutrophil counts decrease after moderate exercise^16,17^, thus contributing to the anti-inflammatory effects of this kind of exercise. Conversely, exhaustive exercise produces an acute leukocytosis, with neutrophil and lymphocyte mobilization^18,19^. On the other hand, moderate physical activity is capable of increasing phagocytic activity^20,21^ but after an exhaustive exercise, the results are quite controversial because both decreases and increases of macrophage phagocytosis have been reported^22,23^. In addition, intensive training has been linked to an inflammatory profile with an increase in pro-inflammatory macrophage cytokines such as interleukin (IL)-1β and tumour necrosis factor (TNF)-α^24^. Likewise, NK cells can be mobilized and their activity can be altered depending on the intensity and duration of the exercise^25^. In blood, in response to physical exercise, NK cells are rapidly mobilized^26^, but after a very prolonged exercise, blood NK cell counts drop below the pre-exercise levels^27^. However, other authors reported that the proportion of circulating NK cells increases in humans after exhaustive exercise, although these cells seem to belong to a subset with lower cytotoxic activity^28^. Controversial results have also been described by Bigley *et al*.^29^ who observed an increase in NK cell cytotoxicity in trained humans, whereas Campbell *et al*.^30^ showed no differences in NK cell cytotoxic activity in post-menopausal women after 12 months of exercise.

Overall, immune system functions improve with moderate exercise but a training that is too intensive can lead to altered immune functions^31^. In this regard, the alterations to innate immunity due to intensive training are not well established^32^. For this reason, we aimed to analyse the innate immune response in two programmes of intensive training in rats and compare them to that in sedentary animals, as well as to evaluate different time points after the longer training. These programmes could represent short and long intense training periods for young humans, although it is difficult to establish the exact time periods because of the different life length between rats and humans. Anyway, the study was aimed at showing those more sensitive parts of the innate immune system that are modified by intense training in order to study strategies (diet or others) that could avoid such impairment.

## Results

### Performance of training programmes

**Figure 1:**
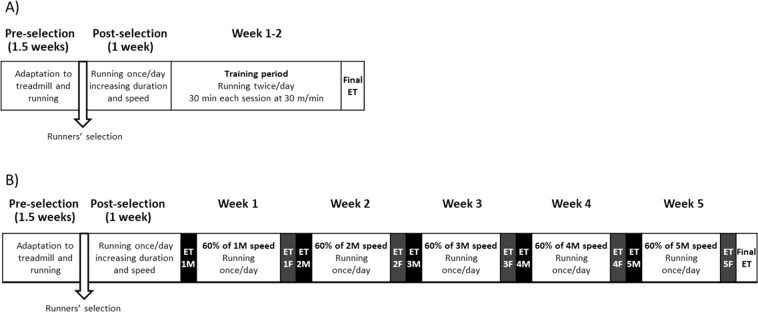
Experimental designs of the training programmes. In the first training programme (**A**), after 2 weeks of short intensive training (weeks 1 and 2), animals ran a final exhaustion test. In the second training programme (**B**), animals were intensively trained for 5 weeks, in which they carried out an exhaustion test every Monday and Friday. An additional final exhaustion test was conducted at the end. ET = exhaustion test, M = Monday, F = Friday.

**Figure 2:**
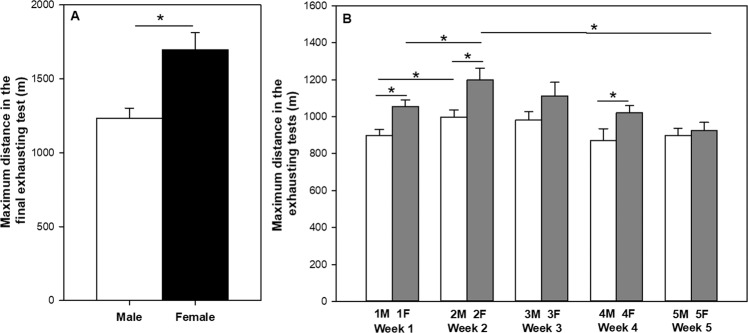
Effect of exercise training on maximum distance run by male and female rats at the final exhaustion test of first training programme (**A**). Effect of exercise training on maximum distance run by female rats throughout the exhaustion tests in the second training programme (**B)**. Data are expressed as mean ± SEM (n = 6–23). ET = exhaustion test, M = Monday, F = Friday. Statistical difference: *p < 0.05 between marked groups (paired –Figure A– and unpaired –Figure B– Student’s t-test).

The first training programme included male and female rats (S-TE groups), which were intensively trained for 2 weeks (twice a day for 30 + 30 min at a speed of 30 m/min, 6 h between them) and afterwards, they carried out a final exhaustion test (Fig. 1). In this final exhaustion test, female rats ran a significantly higher distance (about 1700 m) compared to male runner rats (about 1200 m) (Fig. 2A). Moreover, visual assessment of animals on the treadmill (for example, animals that ran easily) showed that female rats were also better than the males in terms of adaptability to the treadmill. For these reasons, female rats were chosen in the second experimental design.

The second training programme comprised 5-week training with two exhaustion tests plus three trainings per week (Fig. 1). In these exhaustion tests, animals were able to run for 21–25 min at increasing speed, achieving maximum values of 58–72 m/min. The time course of the distance achieved in the exhaustion tests carried out every Monday and Friday is shown in Fig. 2B. In weeks 1, 2 and 4, a longer distance was achieved on Friday when compared to Monday in the same week (p < 0.01 in all three cases). In addition, a longer distance was completed on Monday and Friday of the second week than that on the same day of the first week (p = 0.016 and p = 0.040, respectively). After the second week of training, maximum distance run in the exhaustion tests began to decrease, and the distance achieved on the fifth Friday was shorter than that on the second Friday (p = 0.005). In the additional final exhaustion test, these animals ran a maximum distance of 1419.29 ± 100.03 m (mean ± standard error of the mean or SEM), which was lower than that run by female runner rats of the S-TE group (p < 0.05) in the first training programme.

### Effect of exercise training on body weight and food intake

Male runner rats from the S-TE training did not gain as much weight as male sedentary (SED) rats at the end of the study (p < 0.05) (Table 1). However, no differences in body weight increase were observed between runners and SED female rats in either of the experimental designs. Likewise, we found that exercise training had no influence on food intake.

**Table 1:** Effect of the first training programme (short intensive training or S-TE, lasting 23 days) and the second training programme (intensive training, lasting 55 days) on body weight increase. Data are expressed as mean ± SEM (n = 5–8). Statistical difference: *p < 0.05 versus the corresponding SED group (Student’s t-test).

Training programme	Group	Body weight increase (%)	
		Days 0–23	Days 0–55
Short intensive training	Male sedentary	191.71 ± 5.56	—
	Male runner	149.60 ± 9.01*	—
	Female sedentary	143.65 ± 3.19	—
	Female runner	139.06 ± 5.93	—
**Intensive training**	Female sedentary	144.04 ± 10.80	231.49 ± 17.13
	Female runner	131.78 ± 5.96	226.80 ± 9.79

### Effect of exercise training on blood haemoglobin concentration, haematocrit and leukocyte counts

**Figure 3:**
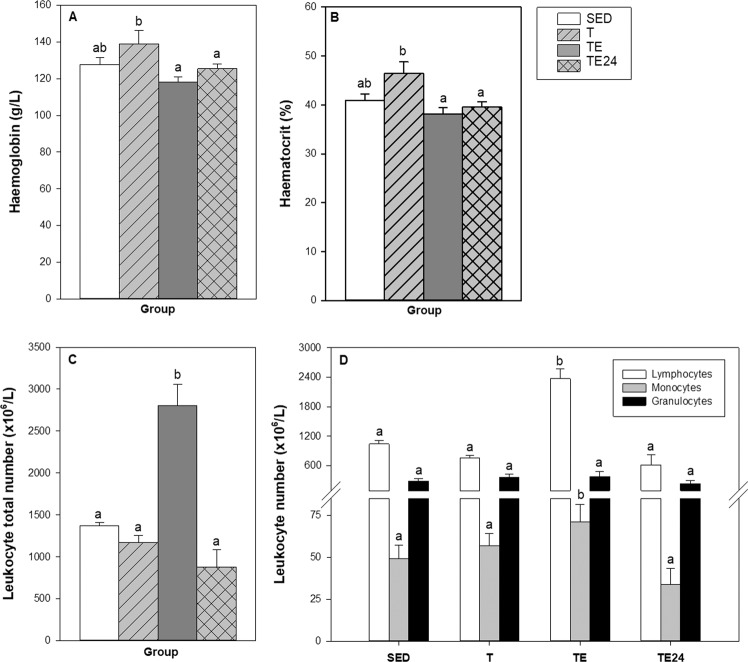
Effect of exercise training on blood haemoglobin concentration (**A**), haematocrit (**B**), number of leukocytes (**C**) and number of lymphocytes, monocytes and granulocytes (**D**) in blood. SED = sedentary rats, T = trained rats, TE = T rats with an additional exhaustion test, and TE24 = TE rats 24 h after the final exhaustion test. Data are expressed as mean ± SEM (n = 7–8). Values not sharing letters denote significant differences (p < 0.05) (Mann–Whitney U test).

In the second experimental design, haemogram data were obtained because previous studies reported that intense exercise modifies white cell counts^33,34^, as well as variables derived from red blood cells^35^. Blood haemoglobin (HGB) concentration and haematocrit (HCT) (Fig. 3A,B, respectively) tended to increase in the T group (p = 0.061) in comparison to the SED group. However, the final exhaustion test (TE group) produced a significant decrease in HGB concentration and HCT compared to the T group (p = 0.005 in both HGB and HCT values), which lasted for at least 24 h (TE24 group) (p = 0.046 and 0.009, respectively).

With regard to the blood leukocyte counts (Fig. 3C), while intensively-trained animals (T group) did not exhibit differences in the total number of leukocytes, the final exhaustion test (TE group) induced a twofold increase in the counts of these blood cells (p < 0.001), due to higher counts of lymphocytes and monocytes, whereas no changes in the number of blood granulocytes was observed (Fig. 3D). One day after the final exhaustion test (TE24 group), the number of leukocytes returned to the physiological counts (Fig. 3C).

**Figure 4:**
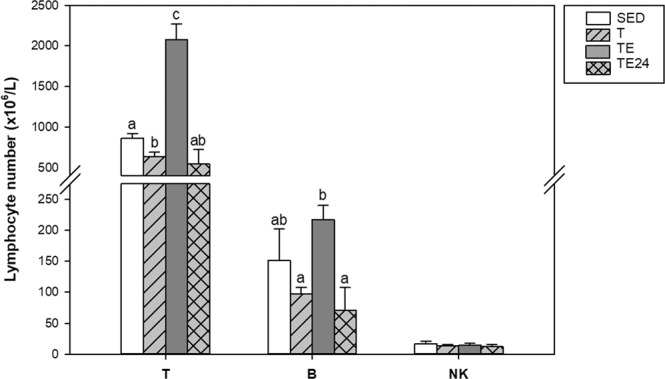
Effect of exercise training on the blood T, B and NK lymphocyte counts. SED = sedentary rats, T = trained rats, TE = T rats with an additional exhaustion test, and TE24 = TE rats 24 h after the final exhaustion test. Data are expressed as mean ± SEM (n = 6–12). Values not sharing letters denote significant differences (p < 0.05) (Mann–Whitney U test).

Considering the main lymphocyte subsets in blood (Fig. 4), intensively trained rats (T group) showed a significant decrease in T-cell counts in comparison with those of the SED animals (p = 0.032). In contrast, the final exhaustion test (TE group) increased the number of blood T and B lymphocytes (p = 0.002 in both cases), which returned to physiological levels 24 h after the final exhaustion test (TE24 group). In the case of NK cells, no changes were detected among the groups (Fig. 4).

### Effect of exercise training on phagocytic activity

**Figure 5:**
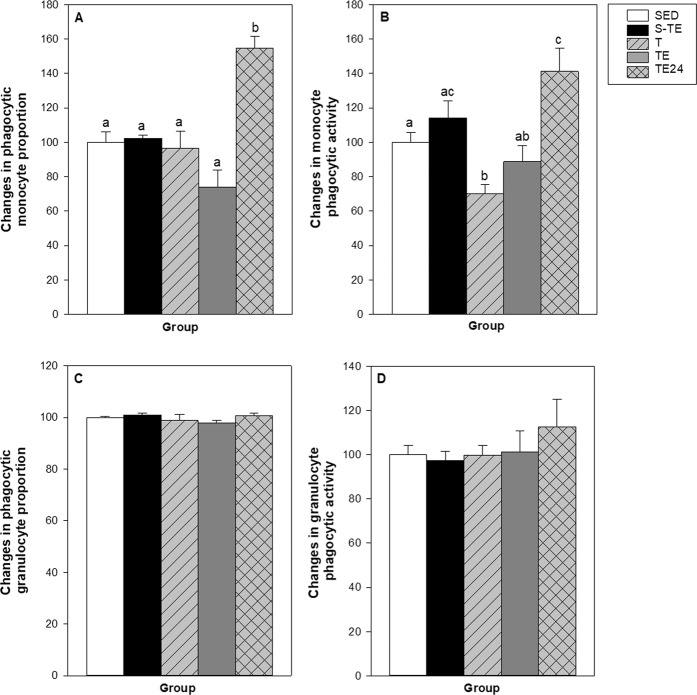
Effect of exercise training on blood phagocytic monocyte proportion (**A**), blood monocyte phagocytic activity (**B**), blood phagocytic granulocyte proportion (**C**) and blood granulocyte phagocytic activity (**D**). SED = sedentary rats, S-TE = rats with a short intensive training and a final exhaustion test, T = trained rats, TE = T rats with an additional exhaustion test, and TE24 = TE rats 24 h after the final exhaustion test. Data are expressed as mean ± SEM (n = 6–12). Values not sharing letters denote significant differences (p < 0.05) (one-way ANOVA).

The proportion of phagocytic monocytes and granulocytes and their phagocytic activity were quantified in blood samples (Fig. 5). In the SED group, the percentage of blood phagocytic monocytes was 50.04 ± 5.86%, which was considered as 100% to better assess the changes induced by the two training programmes. Phagocytic monocyte proportion increased up to 155% 24 h after the final exhaustion test in trained animals of the second training programme (TE24 group), but no changes were observed beforehand (T and TE groups) (Fig. 5A). With regard to the monocyte phagocytic activity, this was lower in trained animals of the second training programme (T group) than in the SED group (Fig. 5B). However, the TE24 group had a higher phagocytic activity than that in the SED, T and TE groups (Fig. 5B).

With regard to granulocytes, the percentage of phagocytic cells in the SED group was 95.79 ± 0.53% and, similarly to monocytes, this proportion was considered as 100% to better assess changes induced by the two training programmes. No changes were observed in any condition either in the proportion of active cells or in their phagocytic activity (Fig. 5C,D).

### Effect of exercise training on macrophage cytokine production

To shed some light on macrophage functionality, the cytokine pattern secreted by peritoneal macrophages after lipopolysaccharide (LPS) stimulation was established in the second training programme (Table 2). In intensively trained animals (T group), interferon (IFN)-γ and IL-6 concentrations were higher, whereas TNF-α, IL-12 and monocyte chemoattractant protein (MCP)-1 levels were lower than those in SED animals (p = 0.025, p = 0.05, p = 0.027, p = 0.05 and p = 0.038, respectively). On the other hand, the final exhaustion test (TE group) produced changes in the cytokine pattern in comparison to the T group by increasing IL-1β, TNF-α, IL-12, MCP-1 and IL-6 concentrations (p = 0.025, p = 0.014, p = 0.014, p = 0.014 and p = 0.05, respectively), and decreasing IFN-γ and IL-10 levels (p = 0.05 and p = 0.021, respectively). One day later (TE24 group), the levels of IFN-γ, IL-1β, TNF-α, MCP-1 and IL-6 were maintained whereas, the secretion of IL-12 and IL-10 increased (p = 0.027 and p = 0.014, respectively). There was a positive correlation between IL-10 concentration and monocyte phagocytic activity (R = 0.777, p < 0.001).

**Table 2:** Effect of exercise training on the cytokine secreted by LPS-stimulated peritoneal macrophages. SED = sedentary rats, T = trained rats, TE = T rats with an additional exhaustion test, and TE24 = TE rats 24 h after the final exhaustion test. Data are expressed as mean ± SEM (n = 6–8). Values not sharing letters denote significant differences (p < 0.05) (Mann–Whitney U test).

	SED group	T group	TE group	TE24 group
IFN-γ	100 ± 34.26^a^	536.49 ± 4.63^b^	116.69 ± 17.49^a^	115.08 ± 40.19^a^
IL-1β	100 ± 16.97^a,b^	59.36 ± 7.07^a^	118.73 ± 8.36^b^	102.66 ± 6.74^b^
TNF-α	100 ± 16.82^a^	45.60 ± 7.27^b^	106.91 ± 9.36^a^	122.04 ± 21.63^a^
IL-12	100 ± 30.03^a,c^	32.31 ± 4.97^b^	81.42 ± 11.88^a^	171.31 ± 29.99^c^
MCP-1	100 ± 9.91^a^	55.91 ± 2.70^b^	105.98 ± 1.99^a^	107.13 ± 3.23^a^
IL-6	100 ± 19.26^a^	169.81 ± 16.50^b^	242.12 ± 21.24^c^	192.07 ± 31.88^b,c^
IL-10	100 ± 12.93^a,b^	105.37 ± 12.37^a^	62.70 ± 2.39^b^	108.17 ± 10.73^a^

### Effect of exercise training on macrophage reactive oxygen species (ROS) production

Peritoneal macrophage ROS production was also established in the second training programme for 30 and 60 min (Table 3). The final exhaustion test (TE group) significantly increased the ROS synthesis with respect to SED animals (p = 0.0110 and 0.0159 at 30 and 60 min, respectively), but macrophages obtained one day later (TE24 group) showed similar results as those found in the T group.

**Table 3:** Effect of exercise training on the reactive oxygen species (ROS) production by peritoneal macrophages for 60 min. SED = sedentary rats, T = trained rats, TE = T rats with an additional exhaustion test, and TE24 = TE rats 24 h after the final exhaustion test. Data are expressed as mean ± SEM (n = 5). Values not sharing letters denote significant differences (p < 0.05) (one –way ANOVA).

Time	SED group	T group	TE group	TE24 group
30 min	6873 ± 845.8^a^	10015 ± 1325.8^a,b^	11620 ± 1147.4^b^	10287 ± 1221.8^a,b^
60 min	8026 ± 947.0^a^	11593 ± 1412.0^a,b^	13087 ± 1333.1^b^	11904 ± 1453.8^a,b^

### Effect of exercise training on spleen NK proportion and functionality

**Figure 6:**
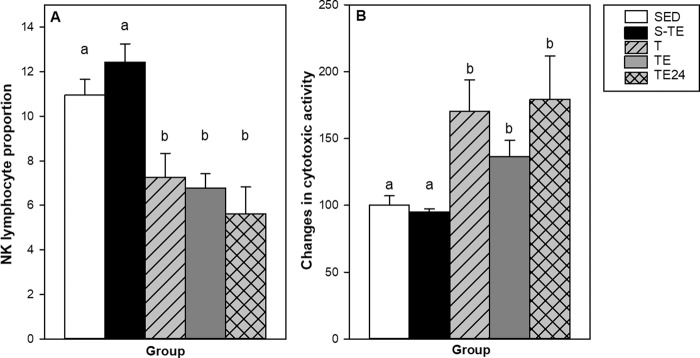
Effect of exercise training on the spleen NK cell proportion (**A**) and the spleen NK cytotoxic individual activity (**B**). SED = sedentary rats, S-TE = rats with a short intensive training and a final exhaustion test, T = trained rats, TE = T rats with an additional exhaustion test, and TE24 = TE rats 24 h after the final exhaustion test. Data are expressed as mean ± SEM (n = 6–12). Values not sharing letters denote significant differences (p < 0.05) (Mann–Whitney U test).

The proportion and functionality of spleen NK cells after both training programmes were established (Fig. 6). The NK cell proportion in the spleen of the SED animals was about 11% (Fig. 6A). No differences were induced by the short training programme (S-TE group), but all conditions from the second training programme exhibited lower NK cell proportions than the SED group and also than the animals from the S-TE group. Interestingly, the spleen NK cell proportion tended to correlate negatively with the total distance run in the final exhaustion test (R = −0.569, p = 0.076), meaning that more distance was accompanied by lower spleen NK cell proportion.

In addition, spleen cytotoxic activity was also modified by exercise. Although the short intensive training programme (S-TE group) did not produce any modification (Fig. 6B), all groups with longer intensive training (T, TE, TE24 groups) markedly increased the spleen NK cytotoxic activity compared to the SED animals (p = 0.007, p = 0.021 and p = 0.039, respectively).

## Discussion

There is no doubt that too intense training can result in an immune system dysfunction that involves a period with higher susceptibility to infectious agents, which is known as the ‘open window’ period^10^. In this study we aimed to analyse the innate immune response in two programmes of intensive training in rats and compare them to that in sedentary rats. For that purpose, two training programmes using a treadmill were applied. A short intensive training for 2 weeks, including two trainings a day (each lasting 30 min) and ending with a final exhaustion test. The second programme included a longer training, lasting 5 weeks and consisting of two exhaustion tests plus three trainings per week, in which samples were collected at the end of the training, immediately after an additional exhaustion test and 24 h after this test.

Firstly, in line with other studies^36–38^, Wistar rats were utilized. Male and female rats were tested in the first training programme, resulting, as expected, in female rats showing a better performance and adaptation than male rats, as previously reported^39,40^. Accordingly, only female rats were used in the second training programme after confirming that the impact of exercise on considered variables was not influenced by rat gender. The exhaustion tests performed during the second training programme evidenced that the rats improved their exercise performance in the first 2 weeks. However, the distance on the Friday of week 5 was lower than that of week 2, which, together with the lower distance achieved in the additional final exhaustion test compared with those from the short intensive training, evidenced a decline in physical performance possibly due to intensive training. This procedure was previously demonstrated to increase oxidative stress in lymphoid tissues^41^.

With regard to changes in the innate immune functionality, although the first experimental design programme included 2 weeks of intensive training, comprising running twice a day (30 min/session for 5 days per week), it was not able to modify the analysed innate immune functions when assessed immediately after the final exhaustion test. Further studies must clarify whether this short intensive training showed changes before and/or 24 h after the final exhaustion test. In contrast, the second training programme, which consisted of two exhaustion tests per week (running about 21–25 min and achieving speeds of 58–72 m/min) plus 3 days of running once a day for 5 weeks, altered the innate immune functionality. In this second experimental design, we determined innate immune functions in basal conditions (24 h after a standard training, T group), immediately after an additional exhaustion test (TE group) and 24 h later (TE-24 group) to establish what happened in the recovering period. Concerning phagocytic cells and activity, in the T group (basal trained conditions), we found no changes in the proportion of blood phagocytic monocytes and neutrophils, but there was a lower phagocytic activity in monocytes. Moreover, peritoneal macrophage functionality was altered, as observed with a higher production of IL-6 and IFN-γ, but lower secretion of IL-12, TNF-α and MCP-1. Higher IL-6 production is in line with increased plasma IL-6 concentrations reported in exercise^42,43^. Even though IL-6 has pleiotropic actions^44^, macrophages are the main cell source of this cytokine, which increased during and after exercise due to muscle damage^45^. Our results suggest that peritoneal macrophages by themselves are able to increase IL-6 secretion in intensively trained rats.

On the other hand, macrophages are a heterogeneous cell type able to polarize into M1 and M2 subsets which are associated with inflammatory and anti-inflammatory functions, respectively^15^. M1 macrophages secrete high levels of pro-inflammatory cytokines, such as TNF-α, IL-1, IL-12 and IL-23, and exhibit higher microbicide functions^15,46^. The lower secretion observed in TNF-α and IL-12 could indicate a partial attenuation of M1 macrophage function, which could explain the high susceptibility to infectious diseases reported after high-intensity exercise^47^. Previous studies in intensively-trained rats demonstrated a decrease in peritoneal macrophage functions, such as a reduction in phagocytic functions^22,48^, which are partially in agreement with our results. Moreover, we found that peritoneal macrophages from trained rats secreted lower amounts of the chemokine MCP-1 (CCL-2), which is able to regulate migration and infiltration of monocytes/macrophages^49^. These results are in line with the concentrations measured in human plasma after running or performing resistance exercises^50,51^, and would agree with the current decrease of other proinflammatory cytokines. On the other hand, peritoneal macrophages from trained rats secreted higher amounts of IFN-γ than those from sedentary animals. The secretion of this proinflammatory cytokine by macrophages is due to IL-12 and IL-18 stimulation^52^ and increases M1 activation^53^. The meaning of the higher IFN-γ levels in trained rats remains to be elucidated, but it can be suggested as a mechanism directed at counteracting the decrease in M1 function.

We also assessed what happened in the macrophage functions when these rats performed an additional final exhaustion test, both immediately after the test and 24 h later, in the recovery period. The lower blood phagocytic monocyte activity observed in the T group was restored immediately after the final exhaustion test (TE group). However, 24 h later, there was an increase in both the number and the phagocytic activity of blood monocytes. In fact, in mice running on a treadmill, it has been reported that phagocytic function increases after severe exercise but not after moderate exercise^54^. The enhanced phagocytosis after an exhausting exercise has been related to the influence of glucocorticoids or catecholamines boosting the macrophage function^23^, and can be seen in the upregulation of the surface receptors^54^ as well as by increased cytokine secretion^55,56^. In this regard, immediately after the final exhaustion test, we observed a higher ROS production by macrophages, a result that is in line with previous studies showing oxidative stress in a similar training^41^. Moreover, there was an increase in macrophage IL-1β, TNF-α, IL-12, MCP-1 secretion, which was mainly reduced in the T group whereas IFN-γ and IL-10 release decreased. One day after the exhaustion test, we found an increase in IL-10 and IL-12 production by peritoneal macrophages. Both IL-10 and IL-12 are able to enhance the phagocytosis capacity^15,57,58^, which is partially in line with the positive correlation detected between IL-10 and monocyte phagocytic activity in the current results.

Another aspect of the innate immune system considered is the NK cell function, which was determined by both the NK cell proportion and cytotoxic activity in the spleen. NK cells represent an important component of the innate immunity and are widely considered as an important link between exercise and health status^25^. Although different authors have related changes in both the number and activity in blood NK cells^25,59^, there are not so many publications studying the NK population in the spleen. A decreased proportion of blood NK cells has been described in humans^60^ and a higher blood NK activity has been reported after chronic exercise (reviewed in^61^). Although we did not detect changes in the number of blood NK cells, we found that T, TE and TE24 groups from the second intensive training had a lower proportion of spleen NK cells. However, these cells had a higher cytotoxic activity, thus suggesting a compensatory immune mechanism in order to maintain the cytotoxic activity in the spleen. In agreement with our results, Blank *et al*.^62^ reported higher NK cell activity in the spleen of trained mice, although they did not find changes in their cell proportion. These changes in rats from the second training programme were not detected in the S-TE group from the first training programme, suggesting that spleen NK percentage could be a biomarker of long intensive training.

Finally, when focusing on blood biomarkers such as haemoglobin concentration, haematocrit and leukocyte counts, we found lower haemoglobin concentrations and haematocrit together with leukocytosis due to higher counts of lymphocytes and monocytes immediately after the exhaustion test. The decrease in haemoglobin concentration and haematocrit could be due to the haemodilution produced for the expansion of blood volume associated with physical activity^63^ and might contribute to the decreased performance capacity^64^. Likewise, it is well established that exercise produces leukocytosis^33,34^ with an increase in lymphocyte counts^65^. This fact seems to be due to the catecholamines released by exercise^66^ that act on lymphocyte adrenergic receptors^67,68^, thus promoting lymphocyte mobilization from both the marginal and the spleen compartments. In our study, the lymphocytosis observed in the TE group was due to increased counts of T and B lymphocytes but not NK cells. These results could be explained by the fact that T cells mobilize faster than other lymphocytes from the spleen compartment^69^.

In summary, our study demonstrated that the intensive training and the exhaustion test carried out in female Wistar rats alter the innate immune system. In particular, intensive training for 5 weeks downregulated M1 macrophage function, but an additional exhaustion test increased monocyte phagocytic activity, which may be related to the altered cytokine pattern and ROS production by macrophages. Moreover, intensive training for 5 weeks induced a lower proportion of spleen NK cells with a higher cytotoxic activity. These findings could partially explain the decline of the immune system due to intensive exercise. Nevertheless, the current results must be completed with further studies focused on the acquired immune system and also in even longer and more intense programmes, in order to clearly show an impairment in the innate immune system.

## Methods

### Animals

The experimental procedures used were approved by the Ethical Committee for Animal Experimentation of the University of Barcelona (UB) and the Catalonia Government (CEEA/UB ref. 464/16 and DAAM 9257, respectively) and performed in accordance with the institutional guidelines for the care and use of laboratory animals.

Four-week-old Wistar rats at the beginning of the experiments (n = 22, males and females in the first training programme; and n = 30, females in the second training programme) were purchased from Envigo (United Kingdom). These young rats, also used by other authors^36,70^, were chosen because they would be adults by the end of the training programme. In addition, previous experiments with nutritional interventions were carried out at this age^41^, a period which is sensitive to the immune system enhancement^71^. Animals were housed in the animal facilities of the Faculty of Biology (UB) in polycarbonate cages (2–3 rats per cage) in a controlled environment of temperature and humidity, in a 12/12 h light/dark cycle. Water and food were provided *ad libitum*. Throughout the study, before running, body weight (BW) and food intake were recorded.

In order to calculate the sample size, the number of animals in each group was established by the Appraising Project Office’s programme from the Universidad Miguel Hernández de Elche (Spain), which allowed the detection of statistically significant differences among groups assuming that there was no dropout rate and a type-I error of 0.05 (two-sided). The variables used in the calculation included the NK activity and the haematological variables, such as the leukocyte cell counts. Moreover, we have adjusted the sample size to the minimum needed by following the University Ethical Committee guidelines and trying to apply the three Rs’ rule for experimenting in animals.

### Training programmes

Two training programmes using a treadmill were applied. In both programmes, two similar devices were used: an LE8700 treadmill (Panlab, Harvard, USA) and an Exer3/6 treadmill (Columbus, Ohio, USA). Both devices, with the same length and width for each running lane, allow the speed and the exercise length to be controlled. In both approaches, sedentary animals and runners were studied in parallel. After running, as a reward, rats received a 50% solution of condensed milk (100 µL/100 g BW); sedentary rats also received this solution.

The exercise training programmes are summarized in Fig. 1. Both experimental designs included 3 days of habituation on a turned-off treadmill, followed by 5 days of preselection in which the treadmills were turned on and rats ran on the treadmill once a day with increasing duration and speed. At the end of this period, sedentary animals (SED group) were randomly selected. During the experiment, the SED group was placed in a treadmill receptacle without movement for the same time as the runners’ rats.

In the first experimental design, i.e., short intensive training with exhaustion (S-TE), the exercise programme was based on a previously described procedure^36^, with some modifications (Fig. 1A). After selection, the runner group began a period in which the duration and the speed was increased progressively (10 min/session at 5 m/min to a 25 min/session at 25 m/min). In the following 2 weeks (weeks 1 and 2), intensive training started and runner animals ran twice a day (30 min at 30 m/min, 6 h between sessions), 5 days per week. At the end of the 2 weeks, animals were subjected to a final exhaustion test, starting with an initial speed of 5 m/min with a gradual increase of 1.8 m/min every min until exhaustion. The animal was considered exhausted when it could not maintain its normal position or when it touched the shock grid more than three times, similarly to that reported in previous studies^72,73^. Immediately after the final exhaustion test, animals were euthanized for sample collection.

The second experimental exercise programme was based on a previously described procedure^74^ (Fig. 1B). After selection, the runner group also began a 1-week period in which the duration and the speed on the treadmill were increased progressively. Afterwards, the intensive training began and lasted for 5 weeks (weeks 1 to 5). In each of these weeks, animals carried out an exhaustion test every Monday and Friday, running 15 min at 60% of the speed of the previous Monday’s exhaustion test (the initial speed of the first Monday’s exhaustion test was 30 m/min), and then the speed was progressively increased, through the device control panel, by 6 m/min every 2 min until rats’ exhaustion. The maximum speed reached on Mondays was used as reference for the three following days, running at 60% of the speed for 20, 25 and 30 min, respectively. Then, the animals were homogeneously distributed into three groups, according to their ability to run: the T group, which was euthanized 24 h after a regular training session, the TE group, which was euthanized immediately after carrying out a final exhaustion test, and the TE24, which was euthanized 24 h after the final exhaustion test to establish the alterations in the recovering period. In the final exhaustion test, animals ran for 15 min at 60% of the speed of the previous Monday’s exhaustion test, and then the speed was increased 3 m/min every 2 min until the animal’s exhaustion.

### Sample collection

Rats were anaesthetized using ketamine (Merial Laboratories S.A., Barcelona, Spain)/xylazine (Bayer A.G., Leverkusen, Germany). Blood, peritoneal macrophages, and spleen were collected and immediately processed. Blood was obtained from the heart in heparin- and EDTA-anticoagulated tubes. An ethylenediaminetetraacetic acid (EDTA)-blood sample was used to determine leukocyte differential count, HGB concentration and HCT (automated haematology analyser, Spincell, MonLab Laboratories, Barcelona). Another EDTA-blood sample was used for characterizing blood lymphocyte composition. Heparinized-blood was devoted to establishing the phagocytic activity. Peritoneal macrophages were used for assessing cytokine secretion and ROS production, as commonly performed in other experimental approaches^75^. From spleen, lymphocytes were isolated to further assess NK cell proportion and cytotoxic activity.

### Determination of phagocytic activity

The phagocytic function of blood monocytes and granulocytes was assessed by flow cytometry analysis using the Phagotest^TM^ kit (Glycotope, Biotechnology GmbH, Heidelberg, Germany) in accordance with the manufacturer’s instructions. Briefly, blood was incubated with fluorescein isothiocyanate (FITC)-labelled *Escherichia coli* (10 min, 37 °C). Then, samples were placed on ice and a quenching solution was added to stop phagocytosis. After washing, erythrocytes were lysed. After DNA staining, data were acquired using Gallios™ Cytometer (CCiTUB) and the analysis was performed with FlowJo v.10 software. Monocyte and granulocyte populations were selected according to their forward-scatter and side-scatter characteristics. The percentage of phagocytic monocytes and granulocytes was quantified by means of FITC+ cells. Their corresponding phagocytic activity was measured through mean fluorescence intensity (MFI). Both the proportion of phagocytic cells and their relative phagocytic activity were compared with the SED group. The data of the corresponding sedentary rats were considered as 100% in each experiment.

### Peritoneal macrophage isolation and stimulation

Peritoneal macrophages were obtained as previously performed^41^. Briefly, after having injected 40 mL of cold phosphate-buffered saline into the peritoneal cavity and after 2 min of abdominal massage, the peritoneal cells were collected. Following centrifugation (538 *g*, 10 min, 4 °C), cells were suspended in cold Roswell Park Memorial Institute (RPMI) medium, supplemented with 10% heat-inactivated foetal bovine serum (FBS), 100 IU/mL streptomycin-penicillin and 2 mM L-glutamine. Cell counts were assessed by a Spincell haematology analyser.

A part of the peritoneal macrophages (10^5^/well) was incubated in RPMI medium without phenol red and supplemented with 1% FBS and were plated into 96-well plates and incubated overnight. After removing non-attached cells (with warm RPMI medium), macrophages were incubated for 30 min with 20 μM of reduced 2’-7’-dichlorofluorescein diacetate probe (H_2_DCF-DA, Invitrogen, Paisley, UK). Macrophage ROS oxidized this probe to a fluorescent compound, which was quantified at 30 and 60 min by a fluorimeter with excitation at 538 nm and emission of 485 nm (Modulus® microplate multimode reader, Turner BioSystems, CA, USA).

Another part of the peritoneal macrophages (10^6^ cells/mL) was plated into 12-well plates and incubated for 2 h. After removing non-attached cells, macrophages were stimulated overnight with 100 ng/mL LPS. Non-stimulated macrophages were included as control. Supernatants were collected for IFN-γ, IL-1β, IL-6, IL-10, IL-12, MCP-1, and TNF-α quantification by using ProcartaPlex® Multiplex Immunoassay (Affymetrix, eBioscience, San Diego, CA, USA), as previously described^71^. Data were acquired by Luminex MAGPIX analyzer (Luminex®) in the Flow Cytometry Unit of the CCiTUB and analyzed with ProcartaPlex® Analyst (Thermo Fisher Scientific, S.L.U, Barcelona, Spain). Results are expressed as percentage considering that the MFI of stimulated macrophages from the SED group was 100%.

### Determination of lymphocyte composition in blood and spleen

To study blood cell changes in depth and also the corresponding cells in the spleen, the main lymphocyte subsets were determined by flow cytometry. After erythrocyte osmotic lysis, blood lymphocyte subsets were determined by mouse anti-rat NKR-P1A, CD45RA, TCRαβ or TCRγδ antibodies (BD Biosciences, San Diego, CA, USA) conjugated to FITC, phycoerythrin or brilliant violet 421, as described previously^76^. Briefly, cells were incubated with the mixture of saturating concentrations of the antibodies (4 °C, 20 min) and, after washing, they were fixed (0.5% p-formaldehyde) and stored (4 °C) until flow cytometry analysis. A blank control was included for each cell sample. Data were acquired with a Gallios™ Cytometer (Beckman Coulter, Miami, FL, USA) in the Flow Cytometry Unit of the Scientific and Technological Centres of the UB (CCiTUB) and analysed with FlowJo v.10 software (Tree Star, Inc., Ashland, OR, USA). Blood lymphocytes are represented as subset counts, taking into account the lymphocyte number from the haematology analyser and the subset percentages obtained by flow cytometry.

To isolate spleen lymphocytes, tissues were smashed in a sterile mesh cell strainer (40 µm) as previously described^76^. After erythrocyte lysis, cells were suspended in culture medium (RPMI medium supplemented with 10% heat- inactivated FBS, 100 IU/mL streptomycin-penicillin, 2 mM L-glutamine and 0.05 mM 2-β-mercaptoethanol, Sigma-Aldrich, Madrid, Spain). NK cell percentage was assessed in spleen suspensions as in blood samples. Changes in the relative proportion of spleen NK lymphocytes are represented considering the SED group value as 100%.

### Assessment of cytotoxic activity by NK cells

The cytotoxic activity of spleen NK cells was quantified by the NKTEST^TM^ kit (Glycotope, Biotechnology GmbH, Heidelberg, Germany) following the manufacturer’s protocol. Briefly, 10^6^ cells/mL splenocytes, containing effector NK cells, were incubated at 37 °C for 1 h to remove monocytes that could suppress NK cell cytotoxic activity. Afterwards, the spleen cells were mixed with target cells (labelled K562 cells) and incubated for 90 min. Immediately before the flow cytometric analysis, a DNA staining was carried out. Data were acquired by the Gallios™ Cytometer (Flow Cytometry Unit, CCiTUB) and the analysis was performed with FlowJo v.10 software. Spontaneous cell death (without effector cells) was considered as control. Results from the individual cytotoxic activity were calculated according to the total NK activity and the percentage of NK cells of each sample. Final results are expressed as changes in the corrected cytotoxic activity considering that the SED rats had 100% of such activity.

### Statistical analysis

Statistical analysis was performed by the IBM Social Sciences Software Program (SPSS, version 22.0, Chicago, IL, USA).

To assess the equality and normality of the results, Levene’s and Shapiro-Wilk tests were used, respectively. Once these conditions were confirmed, a one-way ANOVA test was applied (phagocytic monocyte and granulocyte proportions, phagocytic activities and ROS production). When significant differences were obtained, Bonferroni’s post hoc test was carried out between groups.

Otherwise, non-parametric tests (Kruskal-Wallis test, followed by Mann Whitney U test) were applied to calculate significance (haemoglobin concentration, haematocrit, number of leucocytes, lymphocytes, monocytes and granulocytes in blood, NK cell proportion, cytotoxic activity and macrophage cytokine secretion).

A repeated measures ANOVA test was carried out to assess time-dependent parameters (e.g. BW). When comparing two groups (e.g. males and females, and performance within training), unpaired or paired Student’s t-test was used. According to the equality and normality of the data, a Pearson or Spearman correlation was used to assess correlations between variables. Significant differences were considered when p < 0.05.
